# Development of a highly sensitive enzyme-linked immunosorbent assay (ELISA) through use of poly-protein G-expressing cell-based microplates

**DOI:** 10.1038/s41598-018-36192-8

**Published:** 2018-12-14

**Authors:** Yi-Jou Chen, Michael Chen, Yuan-Chin Hsieh, Yu-Cheng Su, Chang-Hung Wang, Chiu-Min Cheng, An-Pei Kao, Kai-Hung Wang, Jing-Jy Cheng, Kuo-Hsiang Chuang

**Affiliations:** 1 Ph.D. Program in Clinical Drug Development of Chinese Herbal Medicine, Taipei Medical University, Taipei, Taiwan; 2 Center for Biomarkers and Biotech Drugs, Kaohsiung Medical University, Kaohsiung, Taiwan; 3 Department of Biological Science and Technology, National Chiao Tung University, Hsin-Chu, Taiwan; 4 Department of Aquaculture, National Kaohsiung University of Science and Technology, Kaohsiung, Taiwan; 5 Stemforce Biotechnology Co., Ltd, Chiayi City, Taiwan; 6 Center for Reproductive Medicine, Kuo General Hospital, Tainan, Taiwan; 7 National Research Institute of Chinese Medicine, Ministry of Health and Welfare, Taipei, Taiwan; 8 Graduate Institute of Pharmacognosy, Taipei Medical University, Taipei, Taiwan; 9 Ph.D. Program in Biotechnology Research and Development, Taipei Medical University, Taipei, Taiwan; 10 Traditional Herbal Medicine Research Center of Taipei Medical University Hospital, Taipei Medical University, Taipei, Taiwan

## Abstract

The sensitivity of traditional enzyme-linked immunosorbent assays (ELISAs) is limited by the low binding avidity and heterogeneous orientation of capture antibodies coated on polystyrene-based microplates. Here, we developed a highly sensitive ELISA strategy by fixing poly-protein G-expressing cells on microplates to improve the coating amount and displayed orientation of capture antibodies. One or eight repeated fragment crystallisable (Fc) binding domains of protein G are stably expressed on the surface of BALB/c 3T3 cells (termed 1pG cells or 8pG cells), which then act as highly antibody-trapping microparticles. The 8pG cells showed higher antibody-trapping ability than the 1pG cells did. The antibody-coating amount of the 8pG cell-based microplates was 1.5–23 times and 1.2–6.8 times higher than that of traditional polystyrene-based and commercial protein G-based microplates, respectively. The 8pG cell-based microplates were then applied to an anti-IFN-α sandwich ELISA and an anti-CTLA4 competitive ELISA, respectively, and dramatically enhanced their detection sensitivity. Importantly, direct coating unpurified capture antibody produced by mammalian cells did not impair the antigen-capturing function of 8pG cell-based microplates. The 8pG cell-based microplates exhibited a significant improvement in antibody-coating amount and preserved the homogeneous orientation of capture antibodies, making them a potential replacement for traditional microplates in various formats of ELISAs.

## Introduction

ELISAs provide a well-known biochemical analytical method for detecting a substance through a specific interaction between an antibody and its antigen^1–5^. Offering the advantages of high specificity, simplicity, stability, and rapid analysis, ELISAs have become a commonly used tool for analyzing proteins, peptides, and small molecules for clinical and research applications^6–12^. However, the capture antibodies coated on traditional polystyrene-based microplates exhibit a disorganized orientation due to the hydrophobic interactions between the antibodies and the polystyrene surface^13,14^. This random display of the capture antibodies coated on traditional polystyrene-based microplate decreases their antigen-capturing avidity, and further limits the detection sensitivity of the assays^15,16^. In addition, current capture antibodies are produced by expression systems or animal ascites, which contain various irrelevant cellular debris and proteins^17–20^. These impurities would compete with the capture antibodies for the limited area of coating sites on traditional polystyrene-based microplates, a phenomenon which might significantly reduce the detection sensitivity of ELISAs due to interference from the impurities^2^. NH2- or COOH- based microplates, which can form stable covalent bonds between its electrophilic groups and NH2-residues (lysine) or COOH-residues (aspartic acid and glutamic acid) of capture antibodies, also face the same problems as above. It is thus necessary to subject the capture antibodies to a purification process, but doing so increases the cost of traditional polystyrene-based microplates.

Various kinds of strategies for coating capture antibodies on microplates have been developed in order to enhance the detection sensitivity of ELISAs, and one of these commercialized techniques involves the use of protein G-based microplates. Protein G is a streptococcal surface protein which can specifically interact with immunoglobulin and has been widely exploited for biotechnological purposes such as antibody purification^21–25^. By relying on the advantages provided by protein G, commercial protein G-based microplates can be directly coated with capture antibodies without additional purification of the antibodies. However, protein G-based microplates are expensive and time-consuming to manufacture due to the complex process for purifying protein G and fixing it on the microplates. Cell-based microplates constitute another type of microplate sometime used for highly sensitive ELISAs; these microplates are produced by fixing cells to the microplates and then directly expressing capture antibodies on the surfaces of those cells^26^. These microplates provide large antigen-trapping areas and capture antibodies with a homogeneous orientation. However, in order to use such microplates to detect a given antigen, a new cell line expressing a specific corresponding antibody must be established, a process which is labor-intensive and costly. Therefore, existing ELISAs could be made more sensitive, convenient, and cost-effective if one could develop a new type of microplate that combines the advantages of protein G-based microplates and antibody-expressing cell-based microplates.

**Figure 1:**
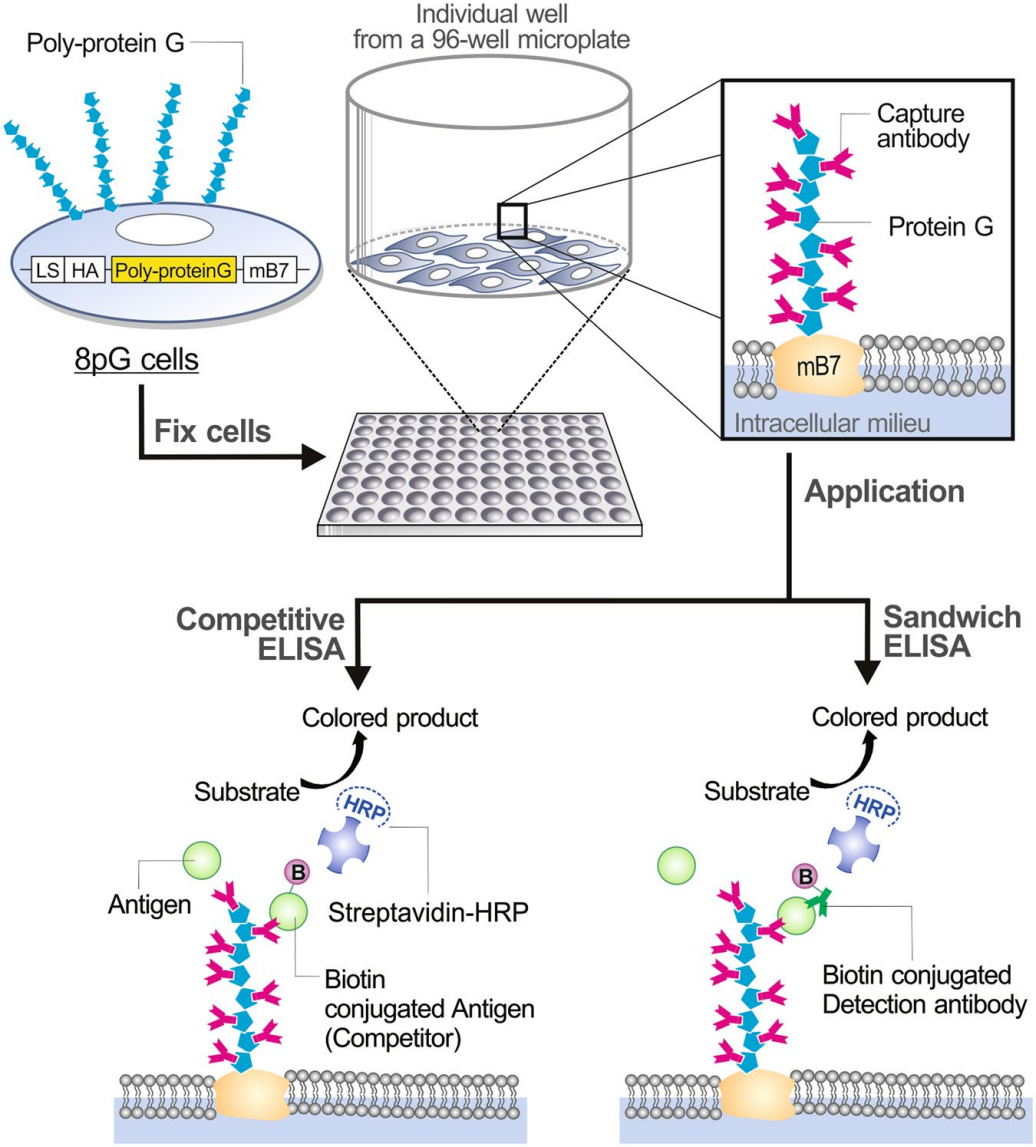
Schematic illustration of poly-protein G cell-based microplate. The poly-protein G gene includes, from N to C terminus, a leader sequence (LS), an HA epitope, the protein G-C2 domain or the eight tandemly repeated protein G-C2 domain, and an immunoglobulin C2-type extracellular-transmembrane-cytosolic domain of the murine B7-1 antigen (mB7). The poly-protein G cell-based microplate utilizes 8pG cells stably expressing poly-protein G on the cell membrane to trap capture antibody. The poly-protein G cell-based microplate can apply to both sandwich ELISAs and competitive ELISAs.

In this study, we developed a novel hybrid microplate for an ELISA with increased detection sensitivity by fixing poly-protein G-expressing cells on the microplate, which then provided a large coating area and homogeneous orientation for any capture antibodies (Fig. 1). The mouse BALB/c 3T3 cells used stably expressed a single or eight tandemly repeated protein G-C2 domains^27^ (the specific binding domain of protein G for immunoglobulin fragment crystallisable (Fc) regions) on their cell surfaces, resulting in cells termed 1pG or 8pG cells, respectively. We assessed the expression and antibody-trapping ability of these 1pG and 8pG cells by western blot and flow cytometry, respectively. The antibody-coating capacity of the 8pG cell-based microplate was compared to that of a traditional polystyrene-based microplate and that of a commercial protein G-based microplate by the induction of biotin-conjugated antibodies. The antigen-capturing ability of anti-CTLA4 antibody coated on these three microplates was compared by capturing the biotin-conjugated soluble ectodomain of CTLA4 (CTLA4-biotin). To assess the performance of a quantitative sandwich ELISA, the anti-interferon-α (IFN-α) antibody/anti-polyethylene glycol (PEG) antibody pairing was used as the capture/detection antibody for detecting PEG-conjugated human IFN-α (Pegasys^®^). In addition, we developed an 8pG cell-based competitive ELISA using CTLA4-biotin to compete with the binding of CTLA4 ectodomain to anti-CTLA4 capture antibody. The performance of unpurified capture antibodies used on the 8pG cell-based microplate was also assessed. The resulting data demonstrated that the antigen-capturing and detection efficiency of the 8pG cell-based microplate were significantly better than those of the traditional polystyrene-based microplate or the commercial protein G-based microplate, suggesting that the 8pG cell-based microplate has the potential to serve as a new generation of highly sensitive microplates for various ELISA formats.

## Results

### Surface expression of functional poly-protein G

**Figure 2:**
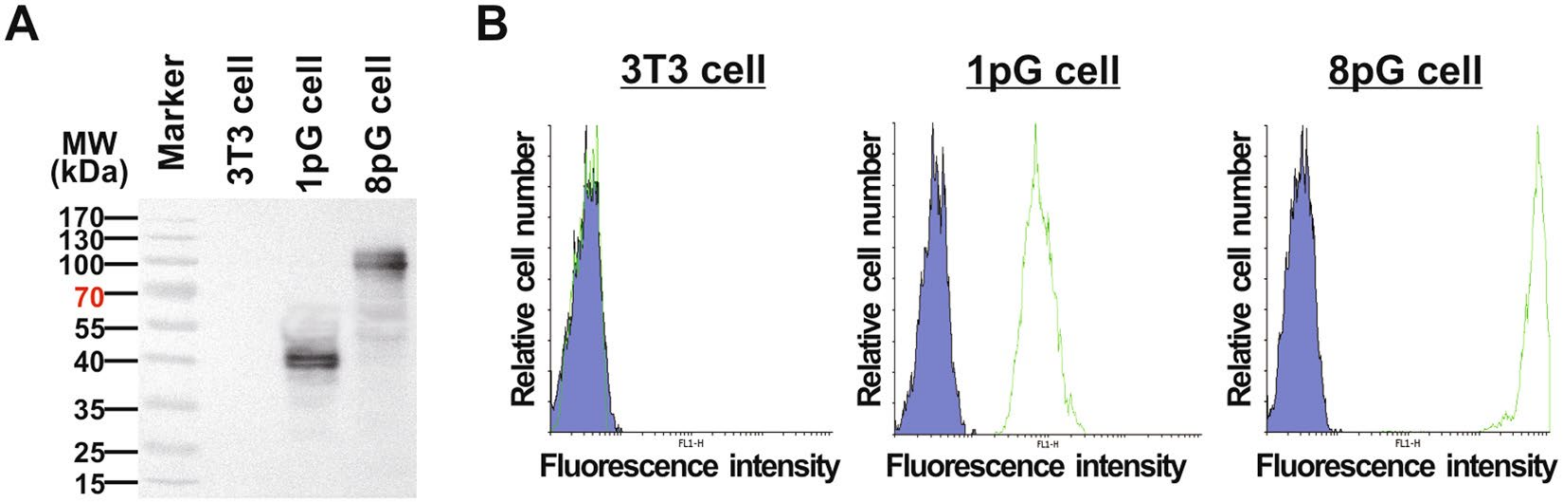
Characterization of poly-protein G-expressing cells. (**A**) The expression of protein G or poly-protein G in BALB/c 3T3 cells was analyzed by western blot using a mouse anti-HA tag antibody. Lane 1, BALB/c 3T3 cells as negative control; Lane 2, single protein G C2 domain expressing BALB/c 3T3 cells (1pG cells); Lane 3, eight tandemly repeated C2 domain expressing BALB/c 3T3 cells (8pG cells). (**B**) The surface displays of functional protein G (middle) and poly-protein G (right) were analyzed by flow cytometry using a FITC-conjugated goat antibody.

The retroviral vectors pLNCX-1pG-mB7 and pLNCX-8pG-mB7 encoded the chimeric proteins in which a single immunoglobulin Fc binding domain (C2 domain) or an eight tandemly repeated C2 domain of protein G was fused to a hemagglutinin (HA) epitope and the immunoglobulin C2-type extracellular-transmembrane-cytosolic domains of mouse B7-1 receptor (mB7) (Fig. 1). Mouse BALB/c 3T3 fibroblasts were infected with the recombinant retroviruses and selected in antibiotic G418 to obtain protein G-expressing cells (1pG cells) and eight repeated protein G-expressing cells (8pG cells). Western blot analysis using anti-HA antibody showed that the cells expressed 1pG and 8pG receptors with the expected size of 38 and 95 kDa, respectively (Fig. 2A). The immunoglobulin trapping ability of the surface 1pG or 8pG was confirmed by flow cytometry after staining the cells with FITC-conjugated goat immunoglobulin. Figure 2B shows that both the 1pG cells and 8pG cells, but not the control 3T3 cells, specifically bound the immunoglobulin. In addition, the fluorescent intensity, which was correlated with the amount of surface-trapped antibody, on the 8pG cells was 52-fold higher than that on the 1pG cells (i.e., a mean fluorescence of 8024.4 *vs*. 158.7). The flow cytometry data indicated that increasing the repeated number of immunoglobulin binding C2 domains on the cell surface dramatically enhanced the antibody-trapping ability of the 8pG cells. Therefore, the 8pG cell line was selected for the following studies.

### Antibody-coating capacity and antigen binding amount on poly-protein G cell-based microplate

**Figure 3:**
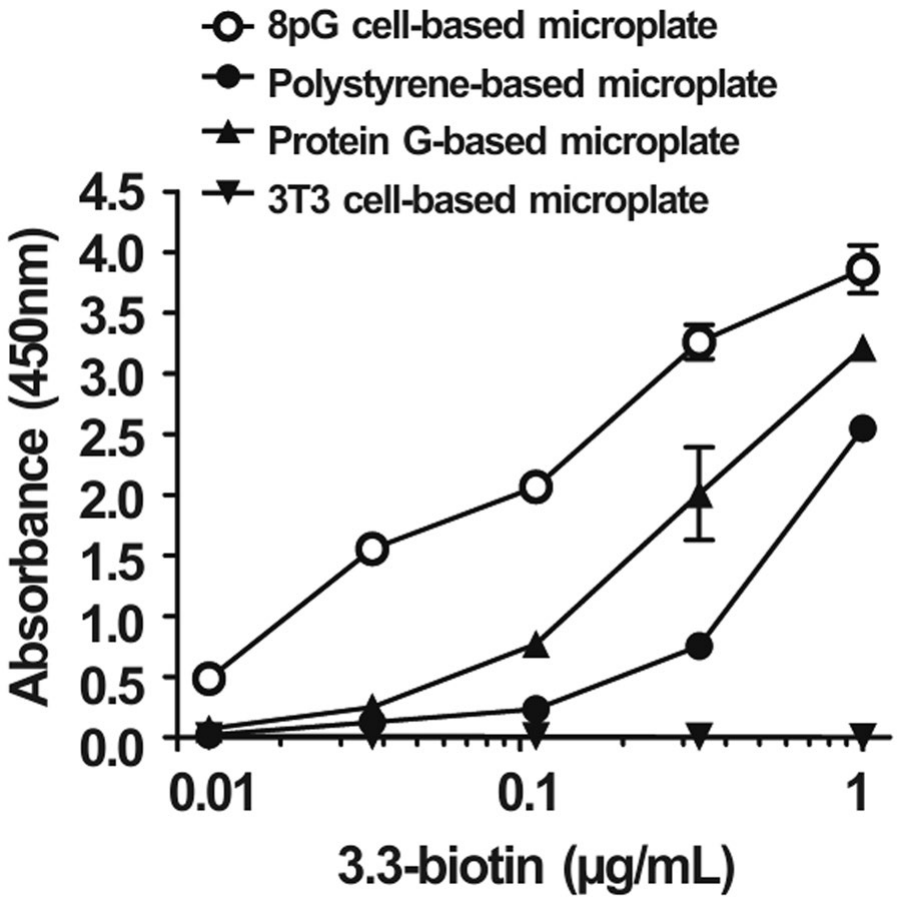
The antibody-coating capacity of poly-protein G cell-based microplate. Graded concentrations of 3.3-biotin were coated on (○) an 8pG cell-based microplate, (●) a traditional polystyrene-based microplate, (▲) a commercial protein G-based microplate, and (▼) a 3T3 cell-based microplate, and detected by streptavidin-HRP. The mean absorbance values (405 nm) of duplicate determinations are shown. Bars, SD.

The antibody-coating capacities of the 8pG cell-based microplate, the traditional polystyrene-based microplate, and the commercial protein G-based microplate were compared by adding different concentrations (0.01, 0.04, 0.11, 0.33, and 1 ug/mL) of biotin-conjugated mouse anti-PEG antibody (3.3-biotin) to each plate, followed by the addition of streptavidin-HRP and 2,2′-azino-bis(3-ethylbenzthiazoline-6-sulfonic acid) (ABTS) substrate. Figure 3 shows that the absorbance as antibody-coating amounts of the 8pG cell-based microplate for those different concentrations were 23-fold (p < 0.01), 12.4-fold (p < 0.001), 8.8-fold (p < 0.001), 4.3-fold (p < 0.001), and 1.5-fold (p < 0.001) higher, respectively, than those of the traditional polystyrene-based microplate, and were 6.8-fold (p < 0.01), 6.2-fold (p < 0.001), 2.7-fold (p < 0.001), 1.6-fold (p < 0.001), and 1.2-fold (p < 0.001) higher, respectively, than those of the commercial protein G-based microplate. These results indicate that the 8pG cell-based microplate offers higher antibody-coating amounts and capacities than the traditional polystyrene-based microplate and the commercial protein G-based microplate.

**Figure 4:**
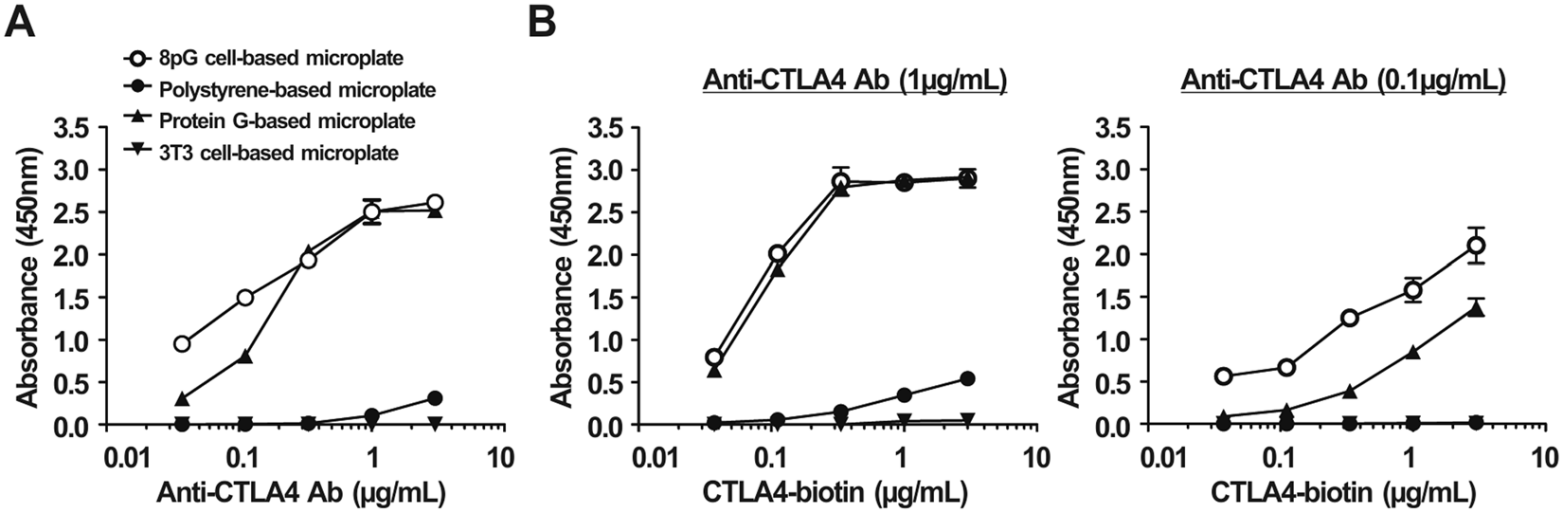
The antigen trapping ability of poly-protein G cell-based microplate. (**A**) Graded concentrations of anti-CTLA4 antibody were coated on different microplates to detect biotin-conjugated CTLA4 (1 μg/ml). (**B**) 1 μg/ml (left) or 0.1 μg/ml (right) concentrations of anti-CTLA4 antibody were coated on different microplates to detect graded concentrations of biotin-conjugated CTLA4. (○) An 8pG cell-based microplate. (●) A traditional polystyrene-based microplate. (▲) A commercial protein G-based microplate. (▼) A 3T3 cell-based microplate. The mean absorbance values (405 nm) of duplicate determinations are shown. Bars, SD.

To further compare the antigen-binding ability of capture antibody on these plates, we added anti-CTLA4 antibody (at concentrations of 0.04, 0.11, 0.33, 1, and 3 ug/mL) into these three plates, followed by the sequential addition of biotin-conjugated CTLA4 ectodomain protein (CTLA4-bition, 1 μg/mL), streptavidin-HRP, and ABTS substrate. Figure 4A shows that the absorbance as antigen-capturing amounts of the 8pG cell-based microplate for those different concentrations of anti-CTLA4 antibody were 728-fold (p < 0.001), 320-fold (p < 0.001), 151-fold (p < 0.001), 24-fold (p < 0.001), and 8.4-fold (p < 0.001) higher than those for the traditional polystyrene-based microplate. Compared with the commercial protein G-based microplate, the 8pG cell-base microplate exhibited no statistical differences in coating the 0.33, 1, and 3 ug/mL concentrations of anti-CTLA4 antibody. However, at the low loading concentrations (0.04 and 0.11 ug/ml) of anti-CTLA4 antibody, the 8pG cell-based microplate captured CTLA4-bition at amounts 3.1-fold (p < 0.001) and 1.9-fold (p < 0.001) greater than those for the commercial protein G-based microplate. To confirm the detection efficacy of different concentrations of capture antibody coated on the 8pG cell-based microplate and the commercial protein G-based microplate, we used 0.1 or 1 ug/mL concentrations of anti-CTLA4 antibody as the capture antibody to detect serially diluted CTLA4-bition (at concentrations of 0.04, 0.11, 0.33, 1, and 3 ug/mL). Figure 4B shows that the 8pG cell-based microplate and the commercial protein G-based microplate showed no statistical differences in absorbance when coated with the 1 ug/mL concentration of anti-CTLA4 antibody. However, when coated with the lower concentration (0.1 ug/ml) of anti-CTLA4 antibody, the CTLA4-captured amounts of the 8pG cell-based microplate were 6.5-fold (p < 0.001), 4.1-fold (p < 0.001), 3.2-fold (p < 0.001), 1.9-fold (p < 0.001), and 1.5-fold (p < 0.05) greater than those for the commercial protein G-based microplate.

### Development of a sensitive poly-protein G cell-based sandwich ELISA

**Figure 5:**
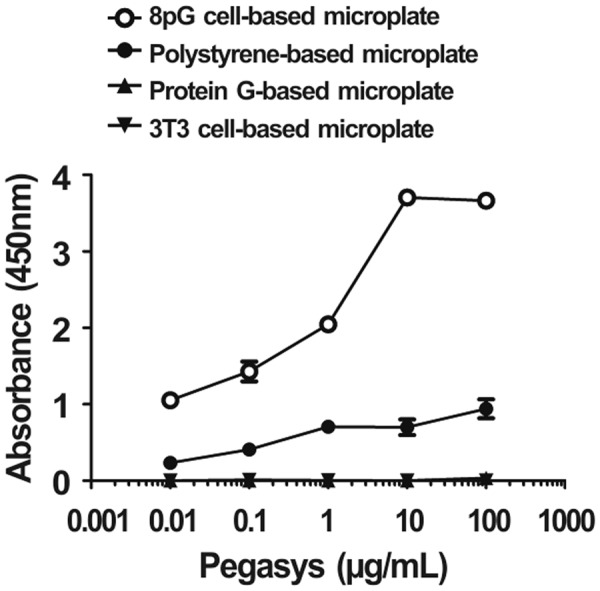
Detection of Pegasys^®^ by poly-protein G cell-based sandwich ELISA. Graded concentrations of Pegasys^®^ were measured by (○) an 8pG cell-based microplate, (●) a traditional polystyrene-based microplate, (▲) a commercial protein G-based microplate, and (▼) a 3T3 cell-based microplate. The mean absorbance values (405 nm) of duplicate determinations are shown. Bars, SD.

We further examined whether the 8pG cell-based microplate could enhance the detection sensitivity of a commercial anti-IFN-α sandwich ELISA kit. After coating the 8pG cell-based microplate, the traditional polystyrene-based microplate, and the commercial protein G-based microplate with anti-IFN-α capture antibody (clone MT1), PEG-conjugated human IFN-α (Pegasys^®^, at concentrations of 0.01, 0.1, 1, 10, and 100 ng/mL) was added into these three plates, followed by the sequential addition of biotin-conjugated anti-PEG IgM antibody (termed AGP3-biotin), streptavidin-HRP, and ABTS substrate. Figure 5 shows that the absorbance amounts of the 8pG cell-based microplate for detecting Pegasys^®^ were 4.5-fold (p < 0.001), 3.5-fold (p < 0.001), 2.9-fold (p < 0.001), 5.3-fold (p < 0.001), and 3.9-fold (p < 0.001) higher than those of the traditional polystyrene-based microplate. However, the commercial protein G-based microplate could not detect Pegasys^®^ effectively in the sandwich ELISA due to the resulting superlative background value (OD 405 is 1.29). The data indicated that the 8pG cell-based microplate, in contrast, could effectively enhance the detection sensitivity of sandwich ELISA kits without increasing the background value.

### Development of a sensitive poly-protein G cell-based competitive ELISA

**Figure 6:**
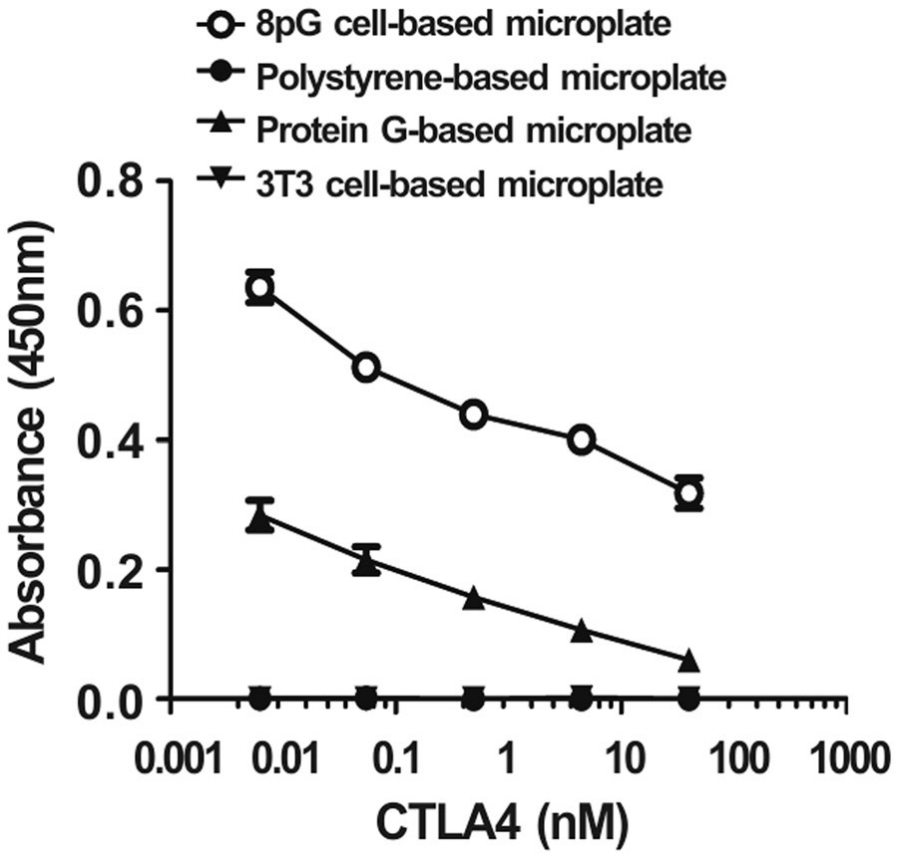
Detection of CTLA4 by poly-protein G cell-based competition ELISA. Graded concentrations of CTLA4 were measured by (○) an 8pG cell-based microplate, (●) a traditional polystyrene-based microplate, (▲) a commercial protein G-based microplate, and (▼) a 3T3 cell-based microplate. The mean absorbance values (405 nm) of duplicate determinations are shown. Bars, SD.

Next, we applied the 8pG cell-based microplate to a competitive ELISA used to measure secreted human CTLA4 antigen. Graded concentrations of CTLA4 were mixed with a fixed amount of CTLA4-biotin (the CTLA4 competitor) prior to addition to the three kinds of microplate coated with 0.1 μg/ml of anti-CTLA4antibody. After extensive washing, the bound CTLA4 competitor was detected with the addition of streptavidin-HRP and ABTS substrate. As shown in Fig. 6, a concentration of CTLA4 as low as 2.5 nM effectively competed with CTLA4-biotin for binding to anti-CTLA4 antibody coated on the 8pG cell-based microplate and the commercial protein G-based microplate. Moreover, the absorbance in the 8pG cell-based microplate was higher than that in the commercial protein G-based microplate, indicating that the 8pG cell-based microplate provides a higher detection sensitivity for competitive ELISAs than the commercial protein G-based microplate does. In contrast, the traditional polystyrene-based microplate coated with 0.1 ug/ml of anti-CTLA4 antibody showed negligible absorbance, indicating that the polystyrene-based microplate is not suitable for competitive ELISAs.

### Unpurified antibody can be used on the poly-protein G cell-based microplate

**Figure 7:**
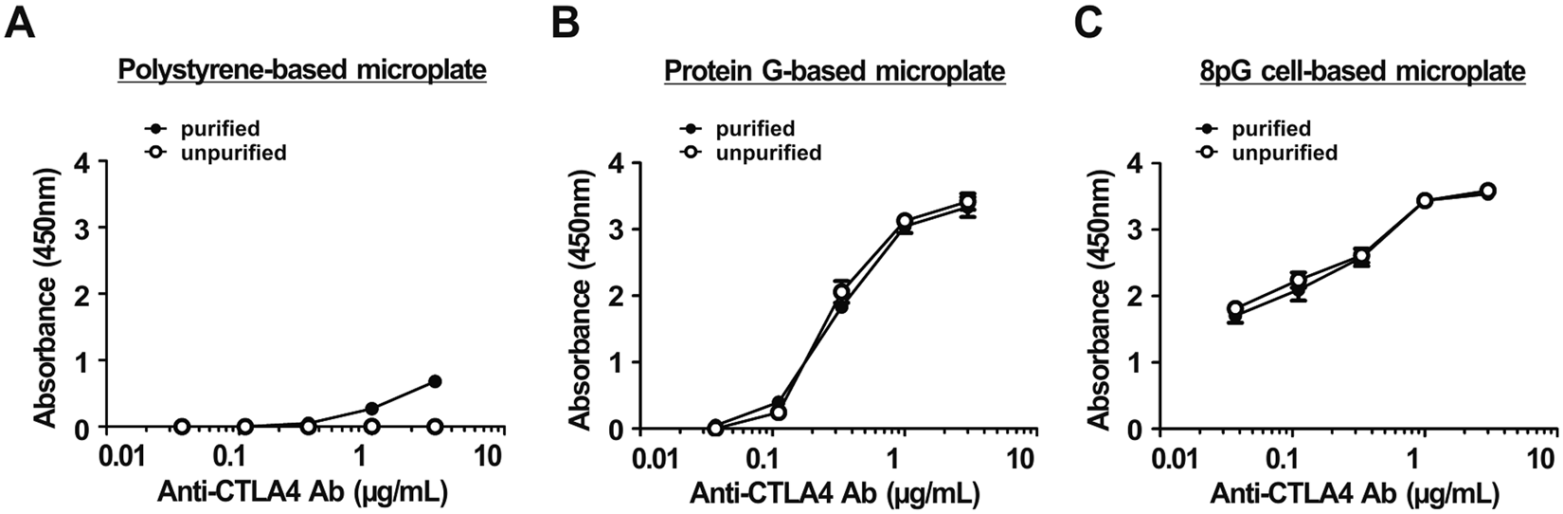
Effect of unpurified capture antibody in poly-protein G cell-based microplate. Graded concentrations of CTLA4-biotin were measured with (●) purified or (○) unpurified anti-CTLA4 capture antibody on (**A**) a traditional polystyrene-based microplate, (**B**) a commercial protein G-based microplate, or (**C**) an 8pG cell-based microplate. The mean absorbance values (405 nm) of duplicate determinations are shown. Bars, SD.

We further tested whether unpurified antibody can be effectively coated on the 8pG cell-based microplate. Samples of purified or unpurified monoclonal anti-CTLA4 antibody produced by mammalian cells were directly coated on the 8pG cell-based microplate, the commercial protein G-based microplate, or the traditional polystyrene-based microplate, and CTLA4-biotin was then added to compare the antigen-capturing amount of these three plates. Captured CTLA4-biotin molecules were quantified by the sequential addition of streptavidin-HRP and ABTS substrate. Negligible coloring signal was shown on the traditional polystyrene-based microplate coated with unpurified anti-CTLA4 antibodies (Fig. 7A and Supplemental Table 1), confirming the necessity of the purification process for traditional polystyrene-based microplates. Conversely, coating purified or unpurified anti-CTLA4 antibody on the commercial protein G-based microplate or the 8pG cell-based microplate did not affect the absorbance (Fig. 7B,C). Moreover, as indicated by the results shown in Fig. 4, the 8pG cell-based microplate coated with anti-CTLA4 antibody exhibited a higher absorbance than the commercial protein G-based microplate for trapping CTLA4-biotin. Collectively, the results indicate that unpurified capture antibodies can be directly coated on the 8pG cell-based microplate, which could dramatically reduce the cost of ELISA kits.

## Discussion

Although ELISAs have been widely used for various medical and biotechnological applications, the sensitivities of traditional polystyrene-based microplates are limited by the low binding avidity and heterogeneous orientation of their capture antibodies. In the present study, we successfully developed an 8pG cell-based microplate in order to increase the amount of capture antibodies coated on the microplate while still preserving the specificity and ensuring a homogenous orientation of the capture antibodies. We demonstrated that the capture antibody-coating capacity and antigen-capturing amount of the 8pG cell-based microplate were higher than those of both a commercial protein G-based microplate and a traditional polystyrene-based microplate. Moreover, when used in both a commercial anti-IFN-α sandwich ELISA kit and an anti-CTLA4 competitive ELISA, the 8pG cell-based microplate showed higher sensitivity than the traditional polystyrene-based microplate and the commercial protein G-based microplate. In addition, unpurified capture antibodies can be directly used on this 8pG cell-based microplate while still exhibiting unimpaired detection ability. The results of our study thus suggest that the 8pG cell-based microplate could serve as a low-cost, convenient, stable, and useful tool for increasing the detection sensitivity of ELISAs (Supplemental Table 2).

The specific immunoglobulin-binding of protein G achieves the optimal coating of capture antibodies on 8pG cell-based microplates. The structure of protein G was first identified by NMR spectroscopy decades ago^24,25,27,28^. The NH2-terminus of protein G binds to human serum albumin, and the COOH-terminus binds to immunoglobulin. The protein G immunoglobulin-binding domain is subdivided into C1, C2, and C3 domains. The C1 and C3 domains bind the antibody Fc and Fab domains, and the C2 domain has been shown by X-ray crystallography to complex with the Fc domain of human IgG^27,29^. As shown in Fig. 5, the commercial protein G-based microplate captured both the immunoglobulin Fab and Fc regions, which resulted in the trapping of IgM type detection antibody (AGP3-biotin) in a sandwich ELISA and the induction of high background noise. Hence, we cloned the protein G-C2 domain to construct the 8pG cell-based microplate, which specifically interacted with the Fc region of IgG type capture antibodies to avoid trapping the IgM type detection antibodies, thereby providing a homogenous orientation of the capture antibodies on the microplate to significantly enhance the detection sensitivity of ELISAs.

Enhancing the detection sensitivity of ELISA microplates is essential for increasing the capture antibody-coated surface area or providing a homogeneous orientation of the capture antibodies. Microplates with high amounts of surface area, such as gold nanoarray-based microplates^30–34^ or polystyrene bead-based microplates^35–37^, provide a greater amount coating area for the capture antibodies, thus enhancing their detection sensitivity. However, the orientation of capture antibodies coated on such microplates is heterogeneous due to the random interaction of the polystyrene surface with any portion of the capture antibodies, and this heterogenous orientation impairs the binding of the capture antibodies and antigens^38^. In our previous study, we expressed a capture antibody on the surface of mammalian cells and coated these cells on a microplate to develop a surface antibody expressing cell-based microplate that possesses various advantages in comparison to traditional polystyrene-based microplates^26,39^. First, the amount of capture antibody immobilized in the cell-based microplate is greater than that immobilized in polystyrene-based microplates due to the larger amount of surface area provided by the cells as compared to a flat-bed well. Second, a capture antibody can exhibit a unidirectional organization (i.e., outward organization) on the surface antibody expressing cell-based microplate, which increases the antigen detection efficacy in comparison to that yielded by traditional polystyrene-based microplates. Nevertheless, the cells expressing a specific antibody on their surface have to be established all over again when detecting a new antigen. This process is laborious and time consuming. In this study, therefore, we combined the advantages of cell-based and protein G-based microplates to construct the 8pG cell-based microplate, which can provide extensive surface area and more steric space for trapping capture antibody than a flat-bed well^40^. The 8pG cells, which can be seen as self-renewing microparticles, are easy to obtain and can provide the preferred orientation of a capture antibody by trapping the Fc domain. Based on these advantages, the 8pG cell-based microplate can enhance the detection sensitivity to a picomolar level.

In the present study, we constructed the 8pG on the surface of mammalian cells and demonstrated that the antibody-trapping amount of these 8pG cells was higher than that of 1pG cells. A similar phenomenon was seen in our previous studies; specifically, poly-protein G-expressing bacteria non-covalently conjugated to any detection antibodies and had a higher antibody-trapping amount than mono-protein G-expressing bacteria^40^. The data showed that the polymer protein G (8pG) had higher avidity for trapping antibodies than monomer protein G (1pG). We speculate that this is the result of the following possible causes. First, the long length of the polymer protein G and the flexible linkers between repeated units result in a larger swing radius for trapping capture antibodies^41,42^. Second, the copolymerization of functional monomers creating synthetic polymers increase their functional affinity; for example, protein purification using a ten-repeated histidine tag has a greater effect than such purification using a six-repeated histidine tag^43,44^. Therefore, we increased the repetition of protein G to enhance the efficiency in terms of trapping antibody, thereby enhancing the capture antibody-coating capacity of the 8pG cell-based microplate.

Currently, the capture antibodies used for ELISAs are subject to purification processes. The most common sources of monoclonal antibodies are ascitic fluids produced by hybridoma and a culture medium of gene-transfected Chinese hamster ovary (CHO) cells^45–48^. The crude antibody solutions collected from these sources are contaminated with a variety of other proteins. Protein A and protein G chromatography are the standard methods that are then used to obtain pure antibodies from these sources in a simple and rapid manner^23,49,50^. In our study, the protein G C2 domain was cloned to construct the poly-protein G (8pG), which was then expressed on cell surfaces to make the 8pG cell-based microplate. Even when using an unpurified antibody, we found that the 8pG cell-based microplate was able to prevent the influence of impurities and to maintain its detection sensitivity, advantages which cannot be achieved by traditional polystyrene-based microplates. Therefore, the capture antibody purification step can be skipped before applying a capture antibody to the 8pG cell-based microplate, effectively reducing the cost of using the 8pG cell-based microplate in comparison to traditional polystyrene-based microplates.

In this study, we developed a poly-protein G cell-based microplate that specifically traps the capture antibody Fc domain to provide a homogeneous capture antibody orientation. Thanks to the eight-repeated protein G expressed on the spherical cell surfaces, the 8pG cell-based microplate provides more surface area and an increased amount of capture antibody coating. The 8pG cell-based microplate can be applied to a variety of ELISAs, including direct ELISAs, sandwich ELISAs, and competitive ELISAs, thus giving it greater applicability than traditional polystyrene-based microplates and commercial protein G-based microplates. Unpurified antibodies can be also used on this 8pG cell-based microplate. Based on these results, we believe that the 8pG cell-based microplate can solve the problems associated with traditional polystyrene-based microplates, thus making the 8pG cell-based microplate more valuable for various clinical and biotechnological applications. Moreover, the 8pG cell-based microplate can couple with detection antibodies which link to different types of signal enhancer, such as colorimetric enzyme^51^, photoelectric nanoparticle^52^, or photoelectrochemical enzyme^53,54^, to further develop ultrasensitive immunoassays.

## Materials and Methods

### Reagents and cells

Lipofectamine 2000, Expi293™ Expression System Kit, Opti-MEM, phosphate-buffered saline (PBS), versene, and penicillin-streptomycin were purchased from Invitrogen (Waltham, MA, U.S.A.). Dulbecco’s modified Eagle’s medium (DMEM), bovine calf serum (BCS), and bovine serum albumin (BSA) were purchased from Sigma-Aldrich (St. Louis, MO, U.S.A.). The restriction enzymes HindIII, ClaI, SfiI, and SalI were purchased from BioLabs, Inc. (Ipswich, MA, U.S.A.). Biotin-conjugated monoclonal mouse anti-PEG antibodies (AGP3-biotin (IgM type) and 3.3-biotin (IgG type)) were provided by Dr. Steve R. Roffler (Academia Sinica, Taipei, Taiwan)^55^. FITC-conjugated goat anti-mouse immunoglobulin G Fcγ antibody, horseradish peroxidase (HRP)-conjugated streptavidin, and HRP-conjugated goat anti-mouse IgG Fcγ antibody were purchased from Jackson Immuno Research Labs (West Grove, PA, U.S.A.).

BALB/c 3T3 cells (American Type Culture Collection, Manassas, VA, U.S.A.) were maintained at 37 °C with 5% CO_2_ and grown in culture medium containing DMEM, 10% BCS, and 100 units/mL of penicillin and streptomycin (Invitrogen, Calsbad, CA, U.S.A.). Expi293 cells were maintained in Expi293 Expression Medium (Invitrogen, Calsbad, CA, U.S.A.) at 37 °C and 120 rpm.

### Plasmid construction of protein G C2 domains

The protein G C2 domains were cloned from streptococcal cDNA. The following primers were used for PCR to clone the protein G C2 domains: Protein G C2 sense: 5′-tgctgcggcccagccggcccatcatcatcatcatcatacttacaaacttgttattaatggtaaaacattgaaa-3′; Protein G C2 antisense: 5′- acctttacagttactgaaggaggcggttcaggatgctaaatcgataa-3′. The PCR fragment was digested with SfiI and ClaI, and then was inserted into a retroviral vector pLNCX vector (Clontech, Mountain View, CA, U.S.A) to form pLNCX-1G. After DNA sequencing, the eight tandemly repeated protein G C2 domain plasmids were constructed using the Tomas Kempe method^56^. The single or eight tandemly repeated protein G C2 domain was digested with SfiI and SalI, and then subcloned into another pLNCX vector containing a hemagglutinin (HA) epitope and the immunoglobulin C2-type extracellular-transmembrane-cytosolic domains of mouse B7-1 receptor (mB7) (termed pLNCX-1pG-mB7 or pLNCX-poly-8pG-mB7).

### Generation of poly-protein G-expressing cells and poly-protein G cell-based microplates

Stable protein G-expressing cells were established by retrovirus infection. pLNCX-1pG-mB7 or pLNCX-8pG-mB7 was cotransfected with pVSVG (Clontech, Mountain View, CA, U.S.A) to GP2−293 cells by Lipofectamine 2000 (Invitrogen). Two days after transfection, the culture medium was filtered, mixed with 8 μg/ml of Polybrene (Sigma-Aldrich), and added into BALB/c 3T3 cells. Infected cells were selected in G418-containing medium and sorted according to anti-HA antibody staining to obtain stable cell lines expressing 1pG or 8pG receptors. The 1pG cell-based microplate or 8pG cell-based microplates were developed using a previously described protocol^57,58^. Briefly, 96-well microplates (Nalge Nunc International, Roskilde, Denmark) were coated with 50 μg/mL poly-D-lysine (Corning, New York, U.S.A.) for 1 hour at 37 °C. After PBS washing, the 1pG cells or 8pG cells were seeded into these microplates at 10^5^ cells/well and cultured for 24 hours. Microplates were washed with DMEM twice and PBS once and then were treated with 1% paraformaldehyde for 3 min at room temperature. The fixation was stopped by addition of 0.1 M glycine for 30 min at room temperature. The microplates were blocked with DMEM containing 0.05% (w/v) BSA (DMEM/BSA) for 1 hour at 37 °C to generate the 1pG cell-based microplate or 8pG cell-based microplates.

### Immunoblot analysis of protein G expression

Cell lysates of 1pG cells and 8pG cells were electrophoresed in 10% reducing sodium dodecyl sulfate polyacrylamide gel electrophoresis (SDS-PAGE) and subsequently transferred onto a nitrocellulose membrane. The blots were blocked in 5% milk in phosphate-buffered saline with Tween 20 (PBST) and incubated with mouse anti-HA monoclonal IgG antibody (1 μg/ml, clone MMS-101R, Biolegend, San Diego, CA, U.S.A). HRP-conjugated goat anti-mouse IgG F(ab’)_2_ antibodies (1 μg/ml) and enhanced chemiluminescence (ECL) substrate (Thermo Fisher Scientific, Waltham, MA, U.S.A.) were used to detect protein signals.

### Analysis of antibody-trapping ability of poly-protein G-expressing cell by flow cytometry

The 1pG cells or 8pG cells were suspended at 2 × 10^5^ cells/tube in PBS buffer containing 0.05% (w/v) BSA (PBS/BSA) and then incubated with FITC-conjugated goat antibody (1 μg/ml diluted in BSA/PBS) for 1 hour. After the removal of unbound antibodies by extensive washing with PBS/BSA, the surface fluorescence of the viable cells was measured by a flow cytometer (BD Biosciences, San Jose, CA, U.S.A.), and fluorescence intensities were analyzed with FlowJo software (Tree Star, Inc., San Carlos, CA, U.S.A.).

### Evaluation of the antibody-coating capacity of 8pG cell-based microplate by ELISA

For the traditional polystyrene-based microplate, PBS containing 2% (w/v) skim milk was used as the sample dilution buffer and PBS was used as the wash buffer. Antibody 3.3-biotin (1 μg/ml) serially diluted in coating buffer (0.1 M NaHCO_3_, pH = 9) was added to Maxisorp 96-well microplates (Nalge Nunc International, Rochester, NY, U.S.A.) for 2 hours at 37 °C. The microplates were blocked with 5% (w/v) skim milk in PBS overnight at 4 °C, followed by the sequential addition of streptavidin-HRP (0.5 μg/ml). The microplates were washed with PBS and bound peroxidase activity was measured by adding 150 μL/well of ABTS solution [0.4 mg/mL, 2,2′-azino-bis(3-ethylbenzthiazoline-6-sulfonic acid) (Sigma-Aldrich), 0.003% (w/w) H2O2, and 100 mM phosphate-citrate, pH 4.0] for 30 min at room temperature. Color development was measured at 405 nm on a microplate reader.

For the cell-based microplate or the commercial protein G-based microplate, DMEM containing 0.05% (w/v) BSA (DMEM/BSA) was used as the sample dilution buffer and DMEM was used as the wash buffer. Antibody 3.3-biotin (1 μg/ml) serially diluted in DMEM/BSA was added to the 8pG cell-based microplates or to the commercial protein G-based microplates at room temperature for 1 hour, followed by the sequential addition of streptavidin-conjugated horseradish peroxidase (streptavidin-HRP, 0.5 μg/ml). The microplates were washed with DMEM and bound peroxidase activity was measured by adding 150 μL/well of ABTS solution for 30 min at room temperature. Color development was measured at 405 nm on a microplate reader.

### Preparation of CTLA4-biotin

The soluble ectodomain of human CTLA4 was cloned from human cDNA. The following primers were used for PCR: Extracellular CTLA4-2 sense: 5′-ctagctagcaagcttgccaccatggagacagacacactcctgctatgggtact-3′; extracellular CTLA4-1 sense:5′-cactcctgctatgggtactgctgctctgggttccaggttccactggtgacaaagcaatgcacgtggcc-3′; extracellular CTLA4 antisense: 5′- ttggcgcgccatcgattcagtggtggtggtggtggtggctgccgccgtcagaatctgggca-3′. The PCR fragment was digested with HindIII and ClaI, and then was inserted into pLNCX vector (Clontech, Mountain View, CA, U.S.A) to form pLNCX-extracellular CTLA4. CTLA4 was obtained by using the Expi293 cell Expression System (Invitrogen, Calsbad, CA, U.S.A.) transfected with pLNCX-extracellular CTLA4 and was purified by nickel columns (GE Healthcare, Chicago, IL, U.S.A.) according to the manufacturer’s instructions. To synthesize CTLA4-biotin, CTLA4 was dialyzed against NaHCO_3_ (0.1 M, pH = 8) and incubated with Sulfo-NHS-LC-Biotin (Thermo Fisher Scientific, Waltham, MA, U.S.A.) at a molar ratio of 1:3 (protein:biotin) for 2 hours. The solution was then incubated with Tris-HCl (1 M, pH = 8) to stop the reaction and dialyzed against PBS to remove any uncoupled biotin. The final concentration of CTLA4-biotin was determined by BCA protein assay (Thermo Fisher Scientific, Waltham, MA, U.S.A.).

### Preparation of anti-CTLA4 antibody

Anti-CTLA4 antibody was cloned from ipilimumab (Yervoy; Bristol-Myer Squibb, New York, NY, U.S.A.). The PCR fragment was digested with SfiI and ClaI, and was then inserted into pLNCX vector (Clontech, Mountain View, CA, U.S.A) to form pLNCX-anti-CTLA4 plasmid. Anti-CTLA4 antibody was generated by Expi293 cells transfected with the plasmid and purified by protein G columns (GE Healthcare, Chicago, IL, U.S.A.) according to the manufacturer’s instructions. The final concentration of anti-CTLA4 antibody was determined by BCA protein assay, and the anti-CTLA4 function was confirmed by ELISA.

### Antigen capturing amount of polyprotein G cell-based microplate

For the traditional polystyrene-based microplate, the anti-CTLA4 antibody (3 μg/ml) serially diluted in coating buffer (0.1 M NaHCO_3_, pH = 9) was added to Maxisorp 96-well microplates for 2 hours at 37 °C. The microplates were blocked with 5% (w/v) skim milk in PBS overnight at 4 °C. The CTLA4-biotin (1 μg/mL) samples serially diluted in 2% skim milk were added to the microplates at room temperature for 1 hour. After washing the microplates, streptavidin-HRP and ABTS were sequentially added to them. Color development was measured at 405 nm on a microplate reader.

For the cell-based microplate or the commercial protein G-based microplate, DMEM/BSA was used as the sample dilution buffer and DMEM was used as the wash buffer. The anti-CTLA4 antibody (3 μg/ml) samples serially diluted in DMEM/BSA were coated on the 8pG cell-based microplates or the commercial protein G-based microplates at room temperature for 1 hour. The CTLA4-biotin (1 μg/mL) samples serially diluted in DMEM/BSA were then added to the microplates at room temperature for 1 hour. After washing the microplates, streptavidin-HRP and ABTS were sequentially added to them. Color development was measured at 405 nm on a microplate reader.

### Poly-protein G cell-based sandwich ELISA

For the traditional polystyrene-based microplate, the anti-IFN-α antibody (clone MT1) (2 μg/ml diluted in PBS) was added to Maxisorp 96-well microplates for 2 hours at 37 °C. The plates were blocked with 3% (w/v) BSA at 37 °C for 2 hours. Pegasys^®^ was 10-fold serially diluted in PBS (pH 7.4) containing 2% (w/v) BSA and then was added to the microplates for 2 hours at room temperature. Biotin-conjugated IgM anti-PEG monoclonal antibody (AGP3-biotin) was 1 μg/mL diluted in PBS (pH 7.4) containing 2% (w/v) BSA and then was added to the microplates for 1 hour at room temperature. After washing, the microplates were sequentially incubated with streptavidin-HRP and ABTS. Color development was measured at 405 nm on a microplate reader.

For the cell-based microplate or the commercial protein G-based microplate, DMEM/BSA was used as the sample dilution buffer and DMEM was used as the wash buffer. The anti-IFN-α antibody (2 μg/ml) samples were coated on the 8pG cell-based microplates or the commercial protein G-based microplates at room temperature for 2 hours. Pegasys^®^ (100 ng/ml) was 10-fold serially diluted and then added to the microplates for 2 hours at room temperature. Biotin-conjugated IgM anti-PEG monoclonal antibody (AGP3-biotin) was added (1 μg/ml) to the microplates and incubated for 1 hour at room temperature. After washing, the microplates were sequentially incubated with streptavidin-HRP and ABTS. Color development was measured at 405 nm on a microplate reader.

### Poly-protein G cell-based competitive ELISA

For the traditional polystyrene-based microplate, the anti-CTLA4 antibody (0.1 μg/ml diluted in coating buffer) was added to Maxisorp 96-well microplates for 2 hours at 37 °C. The plates were blocked with 5% (w/v) skim milk in PBS overnight at 4 °C. CTLA4 was 2-fold serially diluted and mixed 1:1 (v/v) with 50 nM CTLA4-biotin (thus, the final concentration of CTLA4-biotin was 25 nM), and then the mixture was added to the traditional antibody-based microplates at room temperature for 1 hour. After extensive washing, the cells were sequentially incubated with streptavidin-HRP and ABTS.

For the cell-based microplate or the commercial protein G-based microplate, DMEM/BSA was used as the sample dilution buffer and DMEM was used as the wash buffer. The anti-CTLA4 antibody (0.1 μg/ml) was coated on the 8pG cell-based microplates or the commercial protein G-based microplates at room temperature for 1 h. CTLA4 was 2-fold serially diluted and mixed with 50 nM CTLA4-biotin in a volume ratio 1:1 (thus, the final concentration of CTLA4-biotin was 25 nM), and then the mixture was added to the 8pG cell-based microplates or to the commercial protein G-based microplates at room temperature for 1 hour. After extensive washing, the cells were sequentially incubated with streptavidin-HRP and ABTS. Color development was measured at 405 nm on a microplate reader.

### Poly-protein G cell-based direct ELISA by using unpurified capture antibody

For the traditional polystyrene-based microplate, the anti-CTLA4 antibody (3 μg/ml serially diluted in Expi293 conditional medium or coating buffer) was added to Maxisorp 96-well microplates for 2 hours at 37 °C. The microplates were blocked with 5% (w/v) skim milk in PBS overnight at 4 °C, followed by the sequential addition of CTLA4-biotin (1 μg/ml diluted in 2% skin milk), streptavidin-HRP, and ABTS.

For the cell-based microplate or the commercial protein G-based microplate, DMEM/BSA was used as the sample dilution buffer and DMEM was used as the wash buffer. The anti-CTLA4 antibody (3 μg/ml serially diluted in Expi293 condition medium or DMEM/BSA) was coated on the poly-protein G cell-based microplates at room temperature for 1 hour, followed by the sequential addition of CTLA4-biotin (1 μg/ml diluted in DMEM/BSA), streptavidin-HRP, and ABTS. Color development was measured at 405 nm by a microplate reader.

## Electronic supplementary material

Supplementary Information
