# Supplementary material for: Development of a highly sensitive enzyme-linked immunosorbent assay (ELISA) through use of poly-protein G-expressing cell-based microplates
Source: Sci Rep. 2018 Dec 14;8:17868. doi: 10.1038/s41598-018-36192-8 (PMC6294806; doi:10.1038/s41598-018-36192-8)
Supplement: Supplementary file 1 — Supplementary Information [file 41598_2018_36192_MOESM1_ESM.docx]

**Development of a highly sensitive enzyme-linked immunosorbent assay (ELISA) through use of poly-protein G-expressing cell-based microplates**

**Yi-Jou Chen^1^, Michael Chen^1^, Yuan-Chin Hsieh^2^, Yu-Cheng Su^3^, Chang-Hung Wang^1^, Chiu-Min Cheng^4^, An-Pei Kao^5^, Kai-Hung Wang^6^, Jing-Jy Cheng^1, 7^, Kuo-Hsiang Chuang^1, 8, 9, 10, *^**

**Supplementary Information**


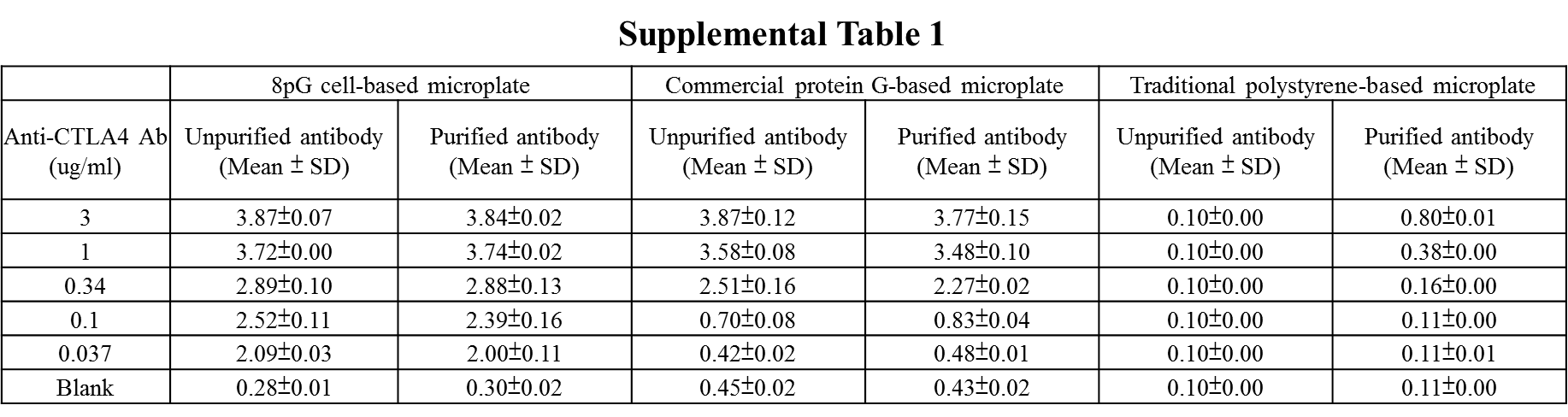


**Supplemental Table 1.** The raw data of an 8pG cell-based microplate, a commercial protein G-based microplate, and a traditional polystyrene-based microplate coated with unpurified or purified anti-CTLA4 capture antibody for the detection of CTLA4-biotin.


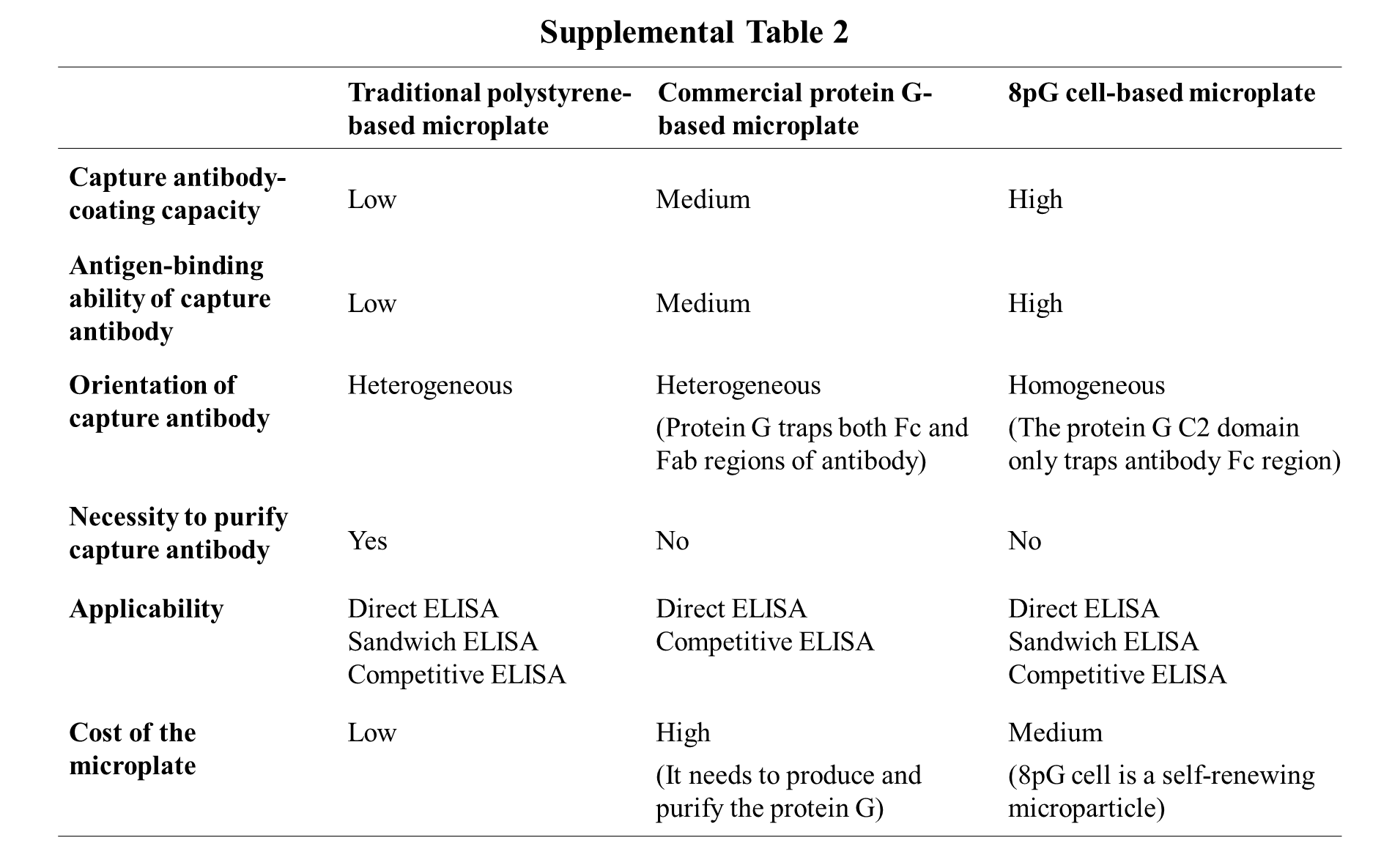


**Supplemental Table 2.** The comparison of the traditional polystyrene-based microplate, the commercial protein G-based microplate and the 8pG cell-based microplate for antibody based ELISA.
